# IMPROVING REPLICABILITY THROUGH DATA STANDARDIZATION: REFINING TELE-STELLA FIDELITY PROCEDURES

**DOI:** 10.1093/geroni/igad104.2567

**Published:** 2023-12-21

**Authors:** Aimee Mooney, Allison Lindauer, Sarah Gothard, Glenise McKenzie, Ruth Tadesse, Hannah Bernard, Charles Boardman, Arlene Danschin

**Affiliations:** Oregon Health & Science University, Portland, Oregon, United States; Oregon Health & Science University, Portland, Oregon, United States; Oregon Health & Science University, Portland, Oregon, United States; Oregon Health & Science University, Portland, Oregon, United States; Oregon Health & Science University, Portland, Oregon, United States; Oregon Health & Science University, Portland, Oregon, United States; Oregon Health & Science University, Portland, Oregon, United States; Oregon Health & Science University, Portland, Oregon, United States

## Abstract

Tele-STELLA (Support via TEchnology: Living and Learning with Advancing Alzheimer’s disease and related dementias) is a prospective, non-randomized, mixed methods clinical trial of a multicomponent videoconference-based psychoeducational intervention for family care partners to facilitate effective management of behavioral symptoms of dementia. The intervention is delivered by nurse “Guides” over two 8-week sessions with the goal of reducing care partner burden, assessed before, during and after intervention. To ensure treatment adherence to this complex intervention protocol, Guides complete intervention checklists after each session. Fidelity monitoring early in study implementation revealed inconsistency in Guide adherence to the intervention protocol as evidenced by gaps and barriers with data collection platforms. To optimize fidelity of the protocol, an iterative process of refinement was applied to procedural operations and data capture practices. These refinements included standardization of protocol tasks (procedural fidelity), explicit definition of guide behaviors (theoretical fidelity) for the continuity of fidelity assessments, and implementation of a unified data capture platform, REDCap®, providing immediate visibility and reference for all team members. We found lower, non-significant Zarit Burden Scores after process revisions (pre=9.14, post=8.43, p=0.29). We also found improvement in data capture with a significantly reduced proportion of missingness for required fields (p=2.2e-16). These findings provide meaningful signaling of improved treatment fidelity, which is associated with those mechanisms of action hypothesized to impact study outcomes. These findings further indicate that a more formal architecture was needed to implement this curriculum and standardization of these processes are vital for long-term replicability and downstream adoption.

